# Attention enhances short‐term monocular deprivation effect

**DOI:** 10.1002/pchj.806

**Published:** 2024-10-13

**Authors:** Jue Wang, Xin He, Min Bao

**Affiliations:** ^1^ CAS Key Laboratory of Behavioral Science, Institute of Psychology Chinese Academy of Sciences Beijing China; ^2^ Department of Psychology University of Chinese Academy of Sciences Beijing China

**Keywords:** attention, binocular rivalry, monocular deprivation, ocular dominance

## Abstract

Patching one eye of an adult human for a few hours has been found to promote the dominance of the patched eye, which is called short‐term monocular deprivation effect. Interestingly, recent work has reported that prolonged eye‐specific attention can also cause a shift of ocular dominance toward the unattended eye though visual inputs during adaptation are balanced across the eyes. Considering that patching blocks all input information from one eye, attention is presumably deployed to the opposite eye. Therefore, the short‐term monocular deprivation effect might be, in part, mediated by eye‐specific attentional modulation. Yet this question remains largely unanswered. To address this issue, here we asked participants to perform an attentive tracking task with one eye patched. During the tracking, participants were presented with both target gratings (attended stimuli) and distractor gratings (unattended stimuli) that were distinct from each other in fundamental visual features. Before and after one hour of tracking, they completed a binocular rivalry task to measure perceptual ocular dominance. A larger shift of ocular dominance toward the deprived eye was observed when the binocular rivalry testing gratings shared features with the target gratings during the tracking compared to when they shared features with the distractor gratings. This result, for the first time, suggests that attention can boost the strength of the short‐term monocular deprivation effect. Therefore, the present study sheds new light on the role of attention in ocular dominance plasticity.

## INTRODUCTION

Ocular dominance plasticity is a classic model to understand experience‐dependent brain plasticity. It has been well known since the 1960s that occluding vision through one eye in an early postnatal period (i.e., the so‐called “critical period”) can lead to long‐lasting anatomical and physiological changes in the visual cortex (Wiesel & Hubel, 1963). Recent work, however, has shown that ocular dominance plasticity is not restricted to the critical period (Lunghi et al., 2011). In Lunghi et al.'s (2011) work, perceptual ocular dominance was measured with binocular rivalry, a task in which two dissimilar but overlapped images, one in each eye, compete for conscious perception. It was found that after 2.5 h of wearing an eye patch, adult participants perceived the image presented to the patched eye more frequently, indicating a shift of ocular dominance toward the patched eye (Lunghi et al., 2011). This effect is thereafter referred to as the short‐term monocular deprivation effect. Unlike ocular dominance plasticity in the critical period, the short‐term monocular deprivation effect can be induced by a relatively brief (15–300 min) period of deprivation (Min et al., 2018), and it can completely decay within 96 min of short‐term monocular deprivation (Min et al., 2018).

In the following decades, the short‐term monocular deprivation effect has gained extensive attention (Binda et al., 2018; Lunghi et al., 2013; Lunghi, Berchicci, et al., 2015; Lunghi, Emir, et al., 2015; Min et al., 2018; Song, Wang, & Bao, 2023; Virathone et al., 2021; Zhou et al., 2014, 2015). The factors modulating this effect have been found to include exercise (but see Baldwin et al., 2022; Finn et al., 2019; Lunghi & Sale, 2015; Virathone et al., 2021; Zhou, Reynaud, & Hess, 2017), fasting (Animali et al., 2023) and dark exposure (Min et al., 2023). Furthermore, the effect is manifested not only in healthy adults (Binda et al., 2018; Lunghi et al., 2013; Lunghi, Berchicci, et al., 2015; Lunghi, Emir, et al., 2015) but also in older children (Nguyen et al., 2023) and adults diagnosed with amblyopia (Lunghi et al., 2019; Zhou et al., 2019).

To further understand the underlying mechanisms behind this type of ocular dominance plasticity, researchers have explored various forms of monocular deprivation other than monocular patching (Bai et al., 2017; Lyu et al., 2020; Ramamurthy & Blaser, 2018; Steinwurzel et al., 2023; Wang et al., 2017; Yao et al., 2017; Zhou et al., 2014). By modifying low‐level visual input, such as energy information (e.g., contrast at a certain range of orientation or spatial frequency) and phase information (e.g., contours) of monocular images, one can also observe a notable shift of ocular dominance toward the deprived eye. Based on these prior findings, the short‐term monocular deprivation effect is believed to mainly originate from the early visual cortex. This notion has also received support from neurophysiological and neuroimaging work, which revealed that the short‐term monocular deprivation effect involves the early visual cortex (Binda et al., 2018; Lunghi, Berchicci, et al., 2015; Zhou et al., 2015), most prominently in primary visual cortex (V1) (Binda et al., 2018).

In addition to the typical short‐term monocular deprivation effect, a surprising form of ocular dominance plasticity has been reported in recent studies on prolonged top‐down eye‐based attention (Song et al., 2024; Song, Lyu, & Bao, 2023; Song, Wang, & Bao, 2023; Wang et al., 2021). To be specific, ocular dominance can be reshaped by solely directing top‐down attention to one eye for 1–1.5 h, during which time visual inputs of both eyes are balanced either by inverting images in one eye with a Porro prism (Wang et al., 2021), or by presenting normal movie episodes to one eye while identical but backward‐played episodes to the opposite eye (Song et al., 2024; Song, Lyu, & Bao, 2023; Song, Lyu, Zhao, & Bao, 2023).

The finding that eye‐based attention itself can reshape ocular dominance opens up new possibilities for research into the influences of high‐level cognitive processing on ocular dominance plasticity. Considering that attention should be biased to the non‐deprived eye during monocular deprivation, an intriguing question thus arises: Can attention also influence the typical short‐term monocular deprivation effect? Unlike typical monocular deprivation that removes all or partial information in one eye, both fundamental features and contour information of visual inputs were preserved and kept equal between the two eyes in the prolonged eye‐based attention studies (Song et al., 2024; Song, Lyu, & Bao, 2023; Song, Lyu, Zhao, & Bao, 2023; Wang et al., 2021). One might argue that the holistic processing of face and biological motion configuration (Sumi, 1984; Tanaka & Farah, 1993) in the unattended eye could be disrupted when using the inverting prism (Wang et al., 2021). However, this is highly unlikely in the dichoptic‐backward‐movie adaptation paradigm (Song, Lyu, & Bao, 2023; Song, Lyu, Zhao, & Bao, 2023). On this basis, the two adaptation paradigms cannot be simply regarded as typical short‐term monocular deprivation. Therefore, the findings in the prolonged eye‐based attention studies may not necessarily provide an answer to the question whether attention influences the typical short‐term monocular deprivation effect.

A recent study had attempted to explore this question (Chen et al., 2020). During monocular patching, their participants were asked to either play action video games with sound, or watch replays of action video games on mute, or play nonaction video games. However, the ocular dominance shift was not significantly different between the three conditions. Thus, they concluded that the short‐term monocular deprivation effect was unaffected by attention. However, caution is needed for this conclusion based on negative results, considering several potential limitations of their methodology. First, there was a lack of direct assessment of attention level in the three conditions. Second, they used a binocular phase combination task rather than a binocular rivalry task to measure ocular dominance. It has been found that the short‐term monocular deprivation effects measured with these two tasks do not always coincide with each other (Bai et al., 2017).

To overcome these potential limitations, here we developed a novel task to control visual attention as much as possible during monocular deprivation. With one eye patched, participants were required to attentively track targets among one of two groups of gratings. The attended gratings and unattended ones had distinct fundamental visual features, which also served as testing gratings in the binocular rivalry tests before and after monocular patching. We hypothesized that when the testing gratings in the pre‐ and post‐tests shared features with the attended gratings during the monocular patching, a more pronounced shift of ocular dominance would be observed if attention could modulate this effect. Moreover, in Chen et al.'s (2020) work, attention level is manipulated across different sessions of monocular deprivation, whereas in the present study, this is achieved within a single session. Our design thus could potentially avoid the perturbation, if any, of the short‐term monocular deprivation effect due to session‐by‐session variation.

## METHODS

### Participants

A total of 40 participants (nine males, aged 22.25 ± 2.47 years) took part in this study. In the short‐term monocular deprivation period, half of the participants wore an eye patch over the dominant eye, and the rest of the participants patched the nondominant eye. To determine the sample size, we first predicted a large effect size (Cohen's *d* = 0.84) according to the results of a previous study on short‐term monocular deprivation (Lyu et al., 2020). Then, we conducted a power analysis using G*Power 3.1 (Faul et al., 2007). Based on an alpha level of .05, a power level of .95, and *d* = .84, the suggested sample size was approximately 21 individuals. All participants had normal or corrected‐to‐normal visual acuity and provided informed consent before the experiment, with no prior knowledge of the experimental hypothesis. The study was in accordance with the ethical standards of the Declaration of Helsinki and approved (H21058, 11/01/2021) by the Institutional Review Board of the Institute of Psychology, Chinese Academy of Sciences.

### Apparatus

The experimental procedure was programmed using the Psychology Toolbox in MATLAB 2021a (Brainard, 1997; Pelli, 1997). The stimuli were presented on a DELL P1230 cathode ray tube (CRT) monitor (1600 × 1200 pixels resolution at the refresh rate of 75 Hz). Prior to the experiment, the CRT monitor was calibrated using a Photo Research PR‐655 photometer, and the mean luminance was 71.87 cd/m^2^. The entire experiment was conducted in a dark room, with participants viewing the stimuli through a mirror stereoscope from a distance of 70 cm. A chin‐rest was used to help minimize head movement.

### Stimuli

#### Binocular rivalry for practice

The stimuli for the binocular rivalry task during the preliminary training period consisted of two orthogonal sinusoidal achromatic gratings (diameter: 1°, spatial frequency: 2 cpd, Michelson contrast: 80%), with the edges of the gratings blurred using Gaussian filtering (standard deviation: 0.7). The orientation of the gratings was either 45° clockwise or counterclockwise. The two gratings were presented to each eye separately at the center of their respective visual fields. To facilitate stable binocular fusion, a central red fixation point (diameter: 0.07°) and a high‐contrast checkerboard frame (size: 2.5° × 2.5°, thickness: 0.25°, Michelson contrast: 0.99) were simultaneously presented to both eyes.

Each trial of the binocular rivalry task lasted for 1 min with a blank screen displayed for the initial 5 s and the grating stimuli presented in the remaining 55 s. Upon the appearance of the grating stimuli, participants were instructed to focus on the red fixation point and press the corresponding key (right, left, or down arrow) on the keyboard based on their perceived orientation of the gratings (clockwise, counterclockwise, or mixed). It is noteworthy that throughout a single trial, the orientations of the gratings presented to both eyes remained consistent, while the grating orientations varied randomly across trials. In our study, the distribution of phase durations approximated a two‐parameter gamma distribution (Figure S1), as consistent with previous work (Levelt, 1967; Lunghi et al., 2013).

#### Binocular rivalry in formal experiment

**FIGURE 1:**
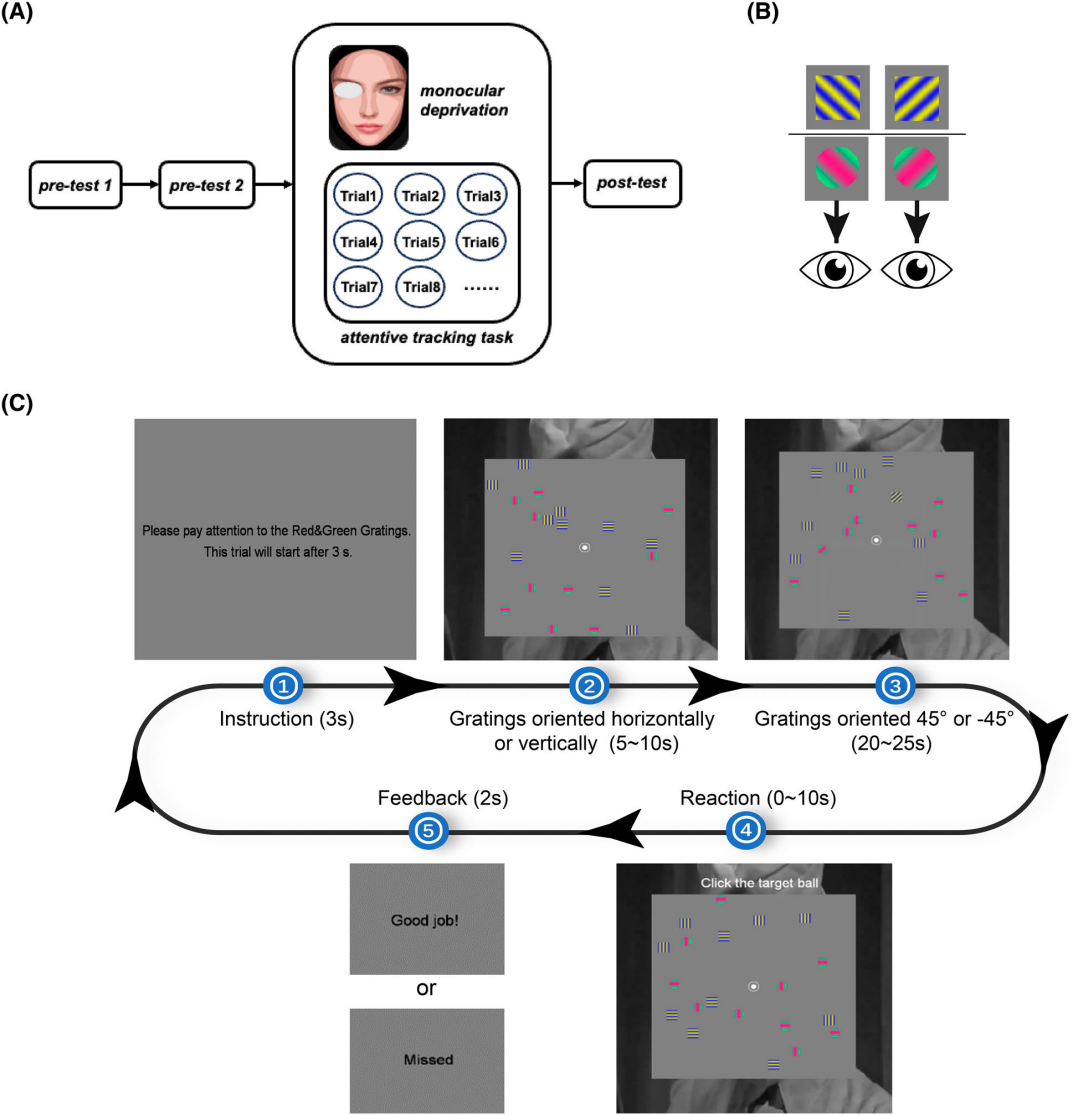
Illustration of the experimental procedure, stimuli and diagram of the tracking task. (A) Schematic diagram of the experimental procedure. (B) Chromatic gratings used in binocular rivalry testing in the formal experiment. (C) Diagram of a trial in the tracking task. Participants were asked to complete the tracking task during monocular deprivation.

In the formal experimental period, the binocular rivalry task served as both the pre‐test and post‐test to measure the changes in ocular dominance. The stimuli consisted of two different types of chromatic orthogonal sinusoidal gratings (see Figure 1A). One was a pair of orthogonally oriented circular red‐green gratings (diameter: 1°, orientation: ±45°, spatial frequency: 1 cpd, Michelson contrast: 80%, phase: 0 or π). The other type of stimulus was a pair of orthogonally oriented square yellow‐blue gratings (diameter: 1°, orientation: ±45°, spatial frequency: 3 cpd, Michelson contrast: 80%, phase: 0 or π). Because the two types of testing gratings differed from each other in various fundamental features (color, shape, and spatial frequency), they might drive relatively nonoverlapping neuronal populations in the V1–V4. Note that these chromatic gratings were not utilized in the binocular rivalry practice period (see *Binocular rivalry for practice*).

Each trial lasted for 1 min, including 5 s of a blank screen followed by 55 s of presentation of grating stimuli. On each trial, both eyes were exposed to either the red‐green grating (a) or the yellow‐blue grating (b). A total of 16 trials were conducted for each binocular rivalry test, with a balanced presentation order of grating stimuli across trials. Specifically, the predetermined order followed either abbabaabbaababba or baababbaabbabaab, thereby resulting in an even distribution of eight trials with the red‐green testing gratings and eight trials with the yellow‐blue ones. To prevent visual aftereffects, the phase values (0 or π) and orientations (±45°) of the grating stimuli remained constant within a trial but were randomly switched between trials. Importantly, the stimuli employed during the post‐test were the same as those used in the corresponding pre‐test, with an identical presentation order.

#### Monocular deprivation

For the purpose of monocular deprivation, an opaque eye patch occluded the participant's one eye (i.e., deprived eye). The deprived eye was the dominant eye (which was determined based on the results of the pre‐test binocular rivalry task) in half of the participants. The light attenuation was 99.88%, and form perception was eliminated by using the eye patch. Throughout the monocular patching, the deprived eye was required to remain open, as recent work has shown that maintaining an open eye during monocular deprivation produces a greater shift in ocular dominance in comparison to keeping the eye closed (Chen et al., 2023).

#### Attentive tracking task

The primary stimuli employed in the tracking task encompassed the chromatic gratings utilized in both the pre‐test and post‐test of the binocular rivalry task (see Figure 1B). There were a total of 10 red‐green (R‐G) gratings and 10 yellow‐blue (Y‐B) gratings. These gratings were presented within an 18° × 18° gray square region, smoothly and independently moving in random directions. To make their movement resemble rigid body motion, they would rebound if colliding with each other, with any border of the square, or with the fixation point (He et al., 2021). Rebounding would occur before the actual contact as if the gratings had a transparent shell with a thickness of 10% diameter, which reduced visual crowding and task difficulty. The rebounding angle was calculated as in a real physical collision, but the speed was controlled to remain constant always. Besides, a gray‐scale image taken from scenes of the television series *iPartment* served as the background surrounding the square to enrich the visual stimuli of the non‐deprived eye.

Tracking consisted of two attention conditions: attending R‐G gratings and attending Y‐B gratings. At the beginning of each session, an instruction appeared on the center of the screen, which prompted the participants to focus their attention solely on the R‐G gratings or Y‐B gratings throughout each trial and ignore the other type of gratings. After 3 s, an 18° × 18° gray square appeared, with 10 R‐G gratings and 10 Y‐B gratings moving from random starting locations in random directions and a gray‐scale image as the background. The gratings were oriented horizontally or vertically. After 5 to 10 s, one R‐G grating and one Y‐B grating would change orientation to 45° or −45°. One of them was the target grating. For instance, in the attending R‐G grating condition, the tilted R‐G grating was designated as the target grating, and participants were instructed to continuously attend to and track the movement of the target grating. After 20 to 25 s, the two gratings tilted back to their original orientations. Meanwhile, a line of instruction appeared above the square, saying “Click the target ball.” The participant then had to click on the current location of the target grating as quickly and accurately as possible within 10 s. Then, the feedback appeared on the screen for 2 s. In the attending Y‐B grating condition, the stimuli were identical to the attending R‐G grating condition, except that the Y‐B grating with tilted orientations was designated as the target grating for tracking.

The duration of each trial of tracking ranged between 35 and 45 s, depending on participants' reaction times. In every 30 trials, participants had a maximum rest period of 20 s. The total duration of the presentation of the chromatic grating on the screen amounted to 1 h. If the duration exceeded 1 h, the program would automatically cease upon completion of the ongoing trial.

### Procedure

Prior to the formal experiment, each participant underwent 3–6 days of binocular rivalry task training. The purpose of this training was to familiarize them with the binocular rivalry task and obtain a stable range for their ocular dominance outcomes (Bao et al., 2018). Each training day consisted of four binocular rivalry tests. The initial one, serving as a 5 min warm‐up, was excluded from data analysis. Subsequently, three tests lasting 16 min each were performed, with a 10 min break between each test. To assess ocular dominance for each participant, an eye ratio index named LvsR was calculated by the formula (*T*_*LE*_ + *T*_*mix*_/2)/(*T*_*RE*_ + *T*_*mix*_/2). In the formula, *T*_*LE*_, *T*_*RE*_, and *T*_*mix*_ represented the summed phase durations for perceiving the stimulus presented to the left eye, right eye, and mixed percepts, respectively. Following our previous work (Bai et al., 2017; Bao et al., 2018), we used this index to assess the stability of the binocular rivalry performance in the practice period and determine the dominant eye. Participants were allowed to advance to the formal experiment if the maximum LvsR value among the three tests did not exceed 110% of the minimum one (which suggested the binocular rivalry performance had been relatively stable).

Each formal experimental day also started with a 5 min warm‐up test of binocular rivalry (achromatic gratings stimuli), followed by a 5 min rest period. Then, two sets of 16 min binocular rivalry pre‐tests (chromatic gratings stimuli) were conducted, with a 10 min break between each set. In each pre‐test, we computed one LvsR based on the data for all 16 trials (i.e., the summed phase duration for each percept was calculated across all trials). Then, the mean LvsR across the two pre‐tests was used to determine the dominant eye of the participant. If the mean LvsR was greater than 1, the participant's left eye was designated as the dominant eye. Otherwise, the right eye was considered the dominant eye. We found that the dominant eye of the majority of participants remained consistent regardless of the stimulus used in binocular rivalry (R‐G or Y‐B). In some participants with a relatively balanced ocular dominance (LvsR close to 1), their dominant eyes could vary depending on the testing stimuli. Consequently, we averaged their LvsR results measured by the two types of gratings across all trials in the two pre‐tests to determine the dominant eye.

Afterwards, participants underwent monocular patching while simultaneously engaging in the tracking task. Upon completion of the tracking task, the eye patch was immediately removed, and a 16 min binocular rivalry post‐test (chromatic gratings stimuli) was conducted. As each participant was required to repeat the experiment twice under each attention condition, they completed a total of four formal experimental sessions, with each session assigned on a different day and the session order counter‐balanced.

### Data analysis

To measure the changes in ocular dominance before and after monocular deprivation, we calculated a metric called Ocular Dominance Index (ODI) using the following formula:

$$\mathrm{ODI}=\frac{T_{\mathrm{DE}}}{T_{\mathrm{DE}}+T_{\mathrm{NDE}}}$$

where *T*_DE_ and *T*_NDE_ represented the summed durations of perceiving stimuli presented to the deprived eye and the non‐deprived eye, respectively. If ODI is larger than 0.5, it means that the deprived eye relatively dominates perception in binocular rivalry.

The ODI for each type of testing gratings in each trial was first calculated. The results for all the trials in each test were then averaged for each participant. Because there were two pre‐tests, the average ODI for the two pre‐tests was used to evaluate the ocular dominance before the deprivation. A 2 (Testing stimuli: R‐G vs. Y‐B grating) × 2 (Attention: unattended vs. attended) × 2 (Time: pre‐ vs. post‐test) repeated analysis of variance (ANOVA) measurements were performed. For the factor of Attention, the “unattended” condition means that the testing gratings shared features with the distractor gratings during the tracking, and the “attended” condition means that the testing gratings shared features with the target gratings. The *post‐hoc* paired *t*‐tests were then conducted to evaluate the monocular deprivation effect for each condition (Bonferroni's correction for multiple comparisons). Furthermore, we calculated the switch rate (number of perception switches per second) as the total switch times divided by the total rivalry time.

## RESULTS

For each participant, the hit rate for the tracking task was calculated by dividing the number of trials with correct responses by the total number of trials. The average hit rate of 74% (SE = 0.02, chance level: 10%, that is, random selecting one out of 10 candidate balls) suggested that the participants focused their attention during the tracking.

**FIGURE 2:**
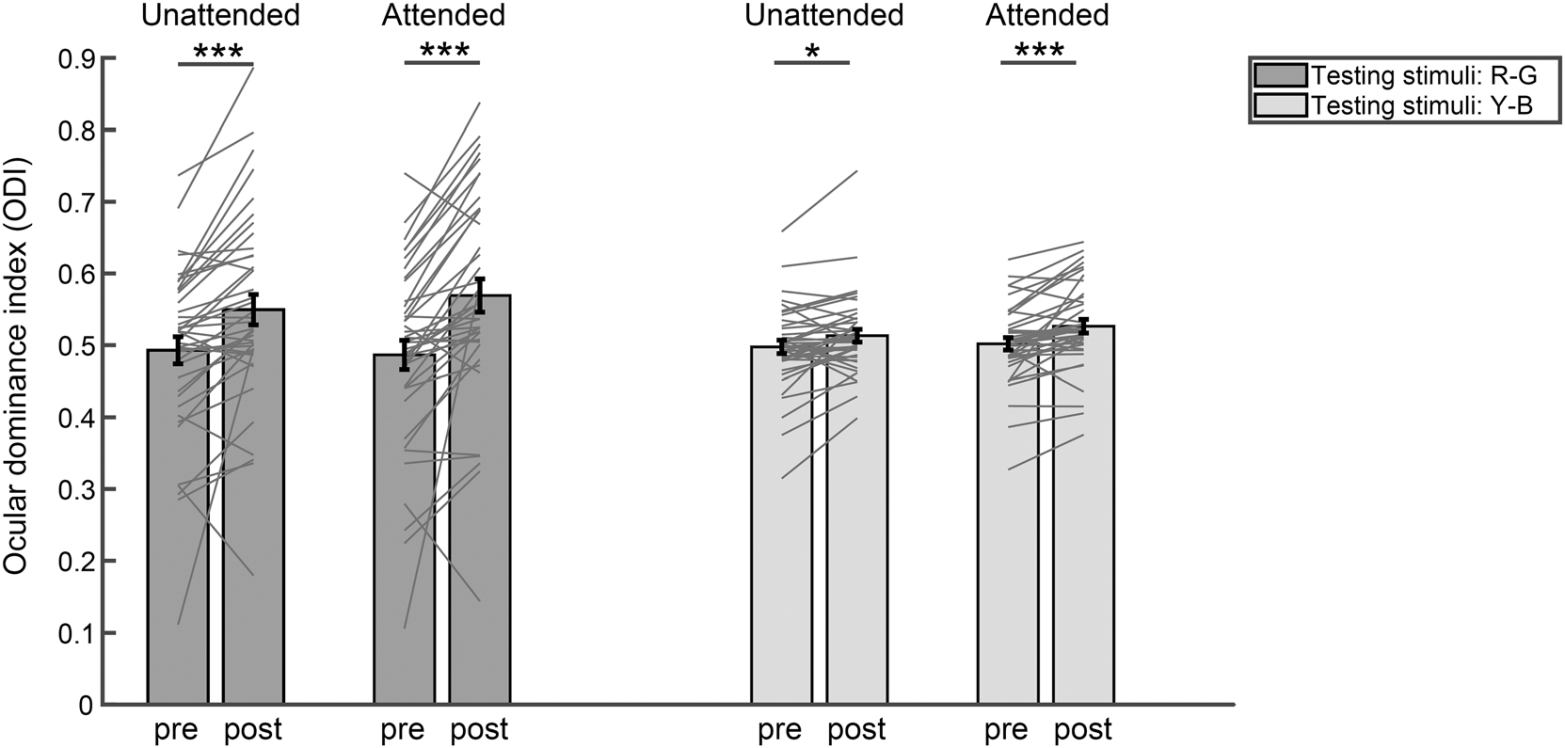
The results of ODI under different attention conditions measured by different testing stimuli in the pre‐ and post‐tests. The bars show the grand average results. The gray lines show the individual data. Error bars represent standard errors of means. Asterisks indicate the significance level of post‐hoc *t‐*test with Bonferroni correction for multiple comparisons (with *p < .05, *** *p* < .001). ODI, ocular dominance index.

To examine whether attention can modulate the short‐term monocular deprivation effect, a 2 (testing stimuli: R‐G vs. Y‐B grating) × 2 (attention: unattended vs. attended) × 2 (time: pre‐test vs. post‐test) repeated ANOVA measurements were conducted on ODI (Figure 2). The results revealed a significant main effect of attention (*F*[1,39] = 5.46, *p* = .025, *η*_*p*_^2^ = 0.12) and time (i.e., the monocular deprivation effect, *F*[1,39] = 25.52, *p* < .001, *η*_*p*_^2^ = 0.40). Further analysis showed that the ODI in the post‐test was higher than that in the pre‐test for both testing stimuli under both unattended and attended conditions (R‐G testing stimuli, Unattended: pre: *M* = 0.49, SE = 0.02, post: *M* = 0.55, SE = 0.02, *p*_bonf_ < .001; R‐G testing stimuli, attended: pre: *M* = 0.49, SE = 0.02, post: *M* = 0.57, SE = 0.02, *p*_bonf_ < .001; Y‐B testing stimuli, unattended: pre: *M* = 0.50, SE = 0.01, post: *M* = 0.51, SE = 0.01, *p*_bonf_ = 0.028; Y‐B testing stimuli, attended: pre: *M* = 0.50, SE = 0.01, post: *M* = 0.53, SE = 0.01, *p*_bonf_ < .001). Importantly, significant two‐way interactions were observed between attention and time (*F*[1,39] = 14.55, *p* < .001, *η*_*p*_^2^ = 0.27), as well as between testing stimuli and time (*F*[1,39] = 23.77, *p* < .001, *η*_*p*_^2^ = 0.38). As shown in Figure 2, the significant attention × time interaction agreed with our expectation that the increment of ODI after deprivation was significantly greater under the two attended conditions (*M* = 0.05, SE = 0.01) compared to the two unattended conditions (*M* = 0.04, SE = 0.01), suggesting an attentional enhancement on the short‐term monocular deprivation effect. Besides, the significant testing stimuli × time interaction suggested that the increment of ODI after deprivation was larger when using R‐G testing stimuli (*M* = 0.07, SE = 0.01) compared to Y‐B testing stimuli (*M* = 0.02, SE = 0.00). Other main effects and interactions were not significant (Table 1).

**TABLE 1:** Statistical results of repeated measurements ANOVA of ocular dominance index.

	Results
Testing stimuli	*F*(1,39) = 1.00, *p* = .324, *η*_*p*_^2^ = 0.03
Attention	*F*(1,39) = 5.46, *p* = .025, *η*_*p*_^2^ = 0.12
Time	*F*(1,39) = 25.52, *p* < .001, *η*_*p*_^2^ = 0.40
Testing stimuli × attention	*F*(1,39) = 0.07, *p* = .795, *η*_*p*_^2^ = 0.00
Testing stimuli × time	*F*(1,39) = 23.77, *p* < .001, *η*_*p*_^2^ = 0.38
Attention × time	*F*(1,39) = 14.55, *p* < .001, *η*_*p*_^2^ = 0.27
Testing stimuli × attention × time	*F*(1,39) = 2.09, *p* = .156, *η*_*p*_^2^ = 0.05

Abbreviation: ANOVA, analysis of variance.

**FIGURE 3:**
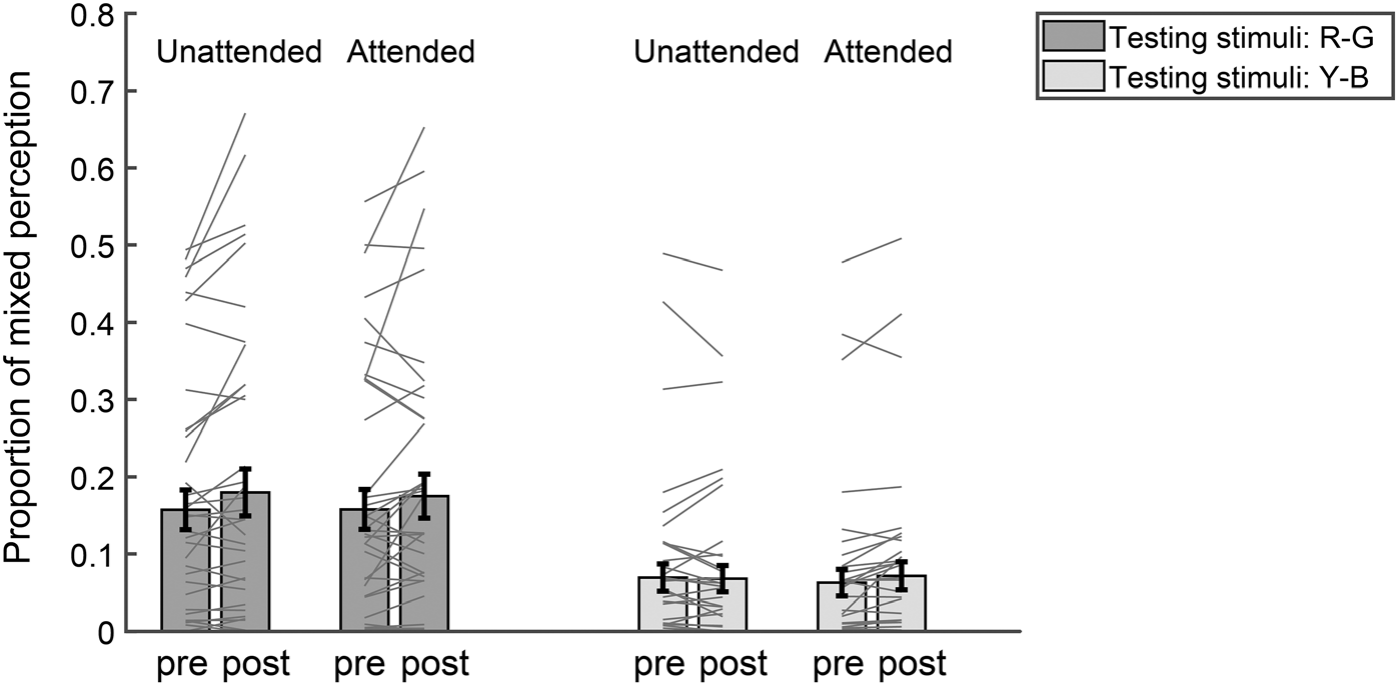
The proportion of mixed percepts under different attention conditions measured by different testing stimuli in the pre‐ and post‐tests. The bars show the grand average results. The gray lines show the individual data. Error bars represent standard errors of means.

To further examine whether attention also influenced the effect of monocular deprivation on mixed perception, we performed 2 (testing stimuli: R‐G vs. Y‐B grating) × 2 (attention: unattended vs. attended) × 2 (time: pre‐test vs. post‐test) repeated ANOVA measurements on the proportion of mixed percepts (Figure 3). The results (Table 2) indicated a significant main effect of testing stimuli with a larger proportion of mixed percepts for the R‐G testing stimuli (*M* = 0.17, SE = 0.01) than for the Y‐B testing stimuli (*M* = 0.07, SE = 0.01). The main effect of time was also significant, showing increased mixed percepts in the post‐test (*M* = 0.12, SE = 0.01) than in the pre‐test (*M* = 0.11, SE = 0.01). Furthermore, there was a significant interaction between testing stimuli and time, showing that following the deprivation, the mixed percepts increased to a larger extent when tested with the R‐G stimuli (pre: *M* = 0.16, SE = 0.02; post: *M* = 0.18, SE = 0.02, *p*_bonf_ = .036) than with the Y‐B stimuli (pre: *M* = 0.07, SE = 0.01; post: *M* = 0.07, SE = 0.01, *p*_bonf_ = .352). Other main effects and interactions were not significant (Table 2).

**TABLE 2:** Statistical results of repeated measurements ANOVA of the proportion of mixed perception.

	Results
Testing stimuli	*F*(1,39) = 21.34, *p* < .001, *η*_*p*_^2^ = 0.35
Attention	*F*(1,39) = 0.35, *p* = .557, *η*_*p*_^2^ = 0.01
Time	*F*(1,39) = 5.81, *p* = .021, *η*_*p*_^2^ = 0.13
Testing stimuli × attention	*F*(1,39) = 0.01, *p* = .930, *η*_*p*_^2^ = 2.03 × 10^−4^
Testing stimuli × time	*F*(1,39) = 5.48, *p* = .024, *η*_*p*_^2^ = 0.12
Attention × time	*F*(1,39) = 0.70, *p* = .408, *η*_*p*_^2^ = 0.02
Testing stimuli × attention × time	*F*(1,39) = 3.13, *p* = .085, *η*_*p*_^2^ = 0.07

Abbreviation: ANOVA, analysis of variance.

**FIGURE 4:**
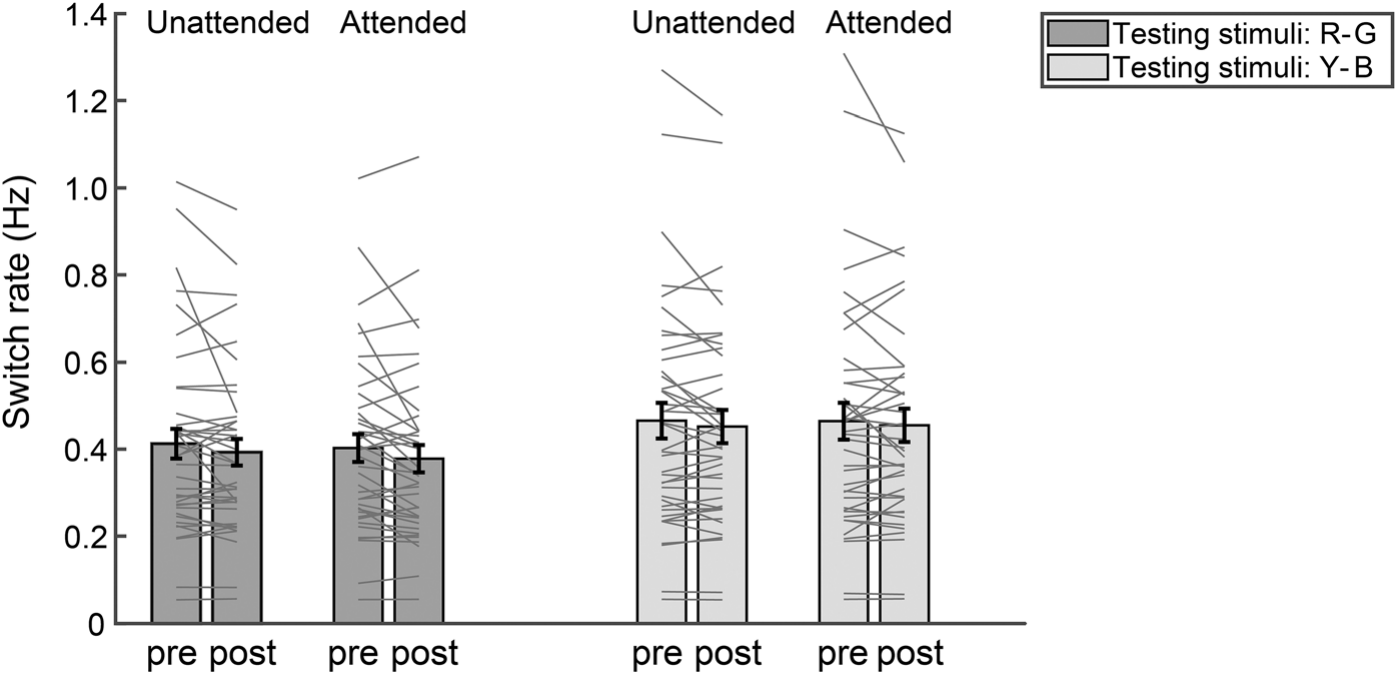
The switch rate under different attention conditions measured by different testing stimuli in the pre‐ and post‐tests. The bars show the grand average results. The gray lines show the individual data. Error bars represent standard errors of means.

To explore whether short‐term monocular deprivation affected switch rate in our study, we also performed 2 (testing stimuli: R‐G vs. Y‐B grating) × 2 (attention: unattended vs. attended) × 2 (time: pre‐test vs. post‐test) repeated ANOVA measurements on the switch rate (Figure 4). The results revealed a significant main effect of testing stimuli that the switch rate was higher when using the Y‐B testing stimuli (*M* = 0.46 Hz, SE = 0.02 Hz) as compared to when using the R‐G testing stimuli (*M* = 0.40 Hz, SE = 0.02 Hz). The main effect of time was also significant, suggesting a slower switch rate in the post‐test (*M* = 0.42 Hz, SE = 0.02 Hz) than in the pre‐test (*M* = 0.44 Hz, SE = 0.02 Hz). Other main effects and interactions were not significant (Table 3). Given that the interaction between attention and time was not significant, the change in switch rate following monocular deprivation was not thought to be influenced by attention.

**TABLE 3:** Statistical results of repeated measurements ANOVA of the switch rate.

	Results
Testing stimuli	*F*(1,39) = 20.10, *p* < .001, *η*_*p*_^2^ = 0.34
Attention	*F*(1,39) = 3.08, *p* = .087, *η*_*p*_^2^ = 0.07
Time	*F*(1,39) = 4.19, *p* = .048, *η*_*p*_^2^ = 0.10
Testing stimuli × attention	*F*(1,39) = 1.73, *p* = .196, *η*_*p*_^2^ = 0.04
Testing stimuli × time	*F*(1,39) = 1.40, *p* = .244, *η*_*p*_^2^ = 0.04
Attention × time	*F*(1,39) = 0.01, *p* = .917, *η*_*p*_^2^ = 2.83 × 10^−4^
Testing stimuli × attention × time	*F*(1,39) = 0.33, *p* = .567, *η*_*p*_^2^ = 0.01

Abbreviation: ANOVA, analysis of variance.

## DISCUSSION

The goal of the present study was to investigate whether attention can modulate the short‐term monocular deprivation effect in adult humans. Our results showed that the post‐test using the attended stimuli (during the tracking) manifested a significantly greater monocular deprivation effect than that using the unattended stimuli. The attended and unattended stimuli in the tracking task differed substantially from each other in visual features (e.g., spatial frequency and color). Thus, the populations of neurons responding to them should be fairly nonoverlapping, though both neuronal populations underwent 1 h of monocular deprivation. The dissociation of the two neuronal populations could be present as early as in V1 (DeBruyn et al., 1993; Engel, 2005). Even if some of the dissociation occurred at higher‐level cortical stages (e.g., V4 or later areas), top‐down attentional feedbacks were still likely to trace back to the monocular neurons that received the corresponding stimuli (Zhang et al., 2012). Therefore, according to the homeostatic compensation theory of ocular dominance plasticity (Lunghi et al., 2013; Turrigiano & Nelson, 2004), the preponderance of activity for the non‐deprived‐eye over the deprived‐eye neurons would be larger in the monocular neurons responding to the attended stimuli than in those responding to the unattended stimuli. This could, in turn, produce a larger monocular deprivation effect in the post‐test using the attended stimuli compared to that using the unattended stimuli.

We also compared the magnitude of the short‐term monocular deprivation effect in the present work with a previous study (Bai et al., 2017) by our lab. By using the same method to calculate the magnitude of the ocular dominance shift, we found that the magnitude of the plasticity effects under the unattended condition in the present work was comparable to that in the previous study (Bai et al., 2017). However, it should be noted that the content and device for visual input between the two studies were not so comparable.

Our finding disagrees with the negative conclusion reached in previous work (Chen et al., 2020). In their work, the level of attentional engagement during patching was varied across sessions, which might introduce session‐by‐session perturbations (if any). Unfortunately, they did not detect significant differences of monocular deprivation effect between attention levels. In the most demanding session, they let participants play action video games, an activity that consumed large attentional resources (Bavelier & Green, 2019). While in the assumed less‐demanding sessions, participants played nonaction video games or merely watched silent replays of video games. However, they did not objectively measure the level of attentional engagement in each session. The attention level in the replay‐watching session is also suspicious, because it is difficult to exclude the possibility that their participants engaged more attention to the silent replay videos than the authors presumed. Indeed, in some cases, human subjects may engage more visual attention when they watch silent movies as compared to when they watch movies with sound (Song, Lyu, Zhao, & Bao, 2023). Another potential factor that may lead to their negative results is that they used the binocular phase combination task for ocular dominance measurements. This task has been shown to mainly probe activities of simple cells in V1 (Huang et al., 2010). The binocular rivalry task we adopted, in contrast, may involve a broader population of neuronal processing (Bai et al., 2017), some of which could be more sensitive to attentional modulations (Tootell et al., 1998).

An unexpected finding of this study is that the deprivation effect tested with R‐G gratings was substantially larger than that tested with Y‐B gratings. This is difficult to explain because, to our knowledge, the chromatic gratings used for binocular rivalry in previous work in this field are either red‐green or red‐blue gratings, rather than yellow‐blue gratings (Animali et al., 2023; Binda et al., 2018; Kurzawski et al., 2022; Lunghi et al., 2013; Lunghi, Emir, et al., 2015; Nguyen et al., 2021; Virathone et al., 2021; Zhou, Reynaud, Kim, et al., 2017). As we know, the parvocellular (P) pathway is highly sensitive to red‐green color contrast, while the koniocellular (K) pathway is specialized for distinguishing yellow‐blue colors (Anssari et al., 2020). Both animal research and monocular deprivation studies on humans have demonstrated greater vulnerability of the P pathway to visual deprivation (Binda et al., 2018; Horton & Hocking, 1997). Therefore, we speculate that short‐term monocular deprivation may exert a stronger impact on the P pathway relative to the K pathway, which may be one of the reasons why a relatively larger deprivation effect was detected using R‐G gratings.

Our results also showed that the proportion of mixed percepts during binocular rivalry significantly increased after monocular deprivation. This result was consistent with the findings of a previous study (Sheynin et al., 2019), which was considered to be due to reduced interocular inhibition. Besides, prolonged periods of binocular rivalry could also cause an increase in the mixed percepts (Klink et al., 2010). However, it should be noted that the interaction of attention × time was nonsignificant, indicating that attention did not exert an obvious influence on the deprivation‐induced interocular disinhibition. Alternatively, the overall low proportion of mixed percepts in our study could lead to an undetectable modulatory effect of attention.

We found a slower switch rate after short‐term monocular deprivation. Previous studies have shown that the shift of ocular dominance after short‐term monocular deprivation is linked to decreased GABA levels in the visual cortex (Lunghi, Emir, et al., 2015). Other work has found that higher GABA concentrations in the visual cortex correspond to slower switch rates (Pitchaimuthu et al., 2017; van Loon et al., 2013). Combining these findings, one may expect that the short‐term monocular deprivation would induce faster switch rates. However, we found the opposite, that is, slower switch rates in the post‐test than in the pre‐test. It should be noted that there is currently no consensus on whether short‐term monocular deprivation causes an increase or decrease in switch rate (Nguyen et al., 2023; Virathone et al., 2021). A previous paper reported no obvious change in switch rate (Virathone et al., 2021), while a subsequent study, like ours, found a significant decrease after monocular deprivation (Nguyen et al., 2023).

In summary, the present study provides evidence supporting the modulatory role of attention in the effect of typical monocular deprivation. Our work suggests that short‐term ocular dominance plasticity is not solely determined by imbalanced visual feedforward inputs but also affected by top‐down attentional feedbacks, discovering potential interplays between higher‐level cognitive functions and lower‐level visual processing in this phenomenon. Some recent studies have found that in older children and adults with amblyopia, inverse occlusion (i.e., patching the amblyopic eye) for multiple daily sessions can effectively improve the visual acuity of the amblyopic eye, and enhance the binocular balance (Lunghi et al., 2019; Zhou et al., 2019). It may be that methods that enhance the magnitude of the short‐term monocular deprivation effect may also enhance the binocular balance in individuals with amblyopia. Increasing attention during monocular patching may be an effective way to increase the monocular deprivation effect in adults with normal vision. Future studies can examine the effects of increasing the visual attentional load during inverse occlusion treatment for amblyopia.

## CONFLICT OF INTEREST STATEMENT

The authors declare no conflicts of interest.

## ETHICS STATEMENT

The study was in accordance with the ethical standards of the Declaration of Helsinki and approved (H21058, 11/01/2021) by the Institutional Review Board of the Institute of Psychology, Chinese Academy of Sciences.

## Supporting information


**Figure S1.** Phase duration distributions are well fitted by a two‐parameter gamma distribution of the form given in Equation (1), categorized by the testing stimuli. The left panel shows the phase duration distribution when using the R‐G testing stimuli, and the right panel displays the phase duration distribution when using the Y‐B testing stimuli. Red, blue, and black curves show the fits to the phase duration distributions for the left eye, right eye, and mixed percepts, respectively.
**Figure S2.** Ocular dominance (LvsR) from two representative participants during the binocular rivalry task training. (a) Participant 1 underwent 3 days of binocular rivalry training. By the third day, the fluctuation of the LvsR values across the three sets of binocular rivalry tasks stabilized within the 10% criterion; thus the participant was deemed eligible to start the subsequent formal experiment. (b) For this participant, the changes of LvsR values for three blocks of binocular rivalry stabilized within 10% on the fourth day. The black dashed line indicates ideally balanced ocular dominance when the LvsR value equals 1.
