# Supplementary material for: Attention enhances short‐term monocular deprivation effect
Source: Psych J. 2024 Oct 13;14(1):84–93. doi: 10.1002/pchj.806 (PMC11787881; doi:10.1002/pchj.806)
Supplement: Supplementary file 1 — Figure S1. Phase duration distributions are well fitted by a two‐parameter gamma distribution of the form given in Equation (1), categorized by the testing stimuli. The left panel shows the phase duration distribution when using the R‐G testing stimuli, and the right panel displays the phase duration distribution when using the Y‐B testing stimuli. Red, blue, and black curves show the fits to the phase duration distributions for the left eye, right eye, and mixed percepts, respectively. Figure S2. Ocular dominance (LvsR) from two representative participants during the binocular rivalry task training. (a) Participant 1 underwent 3 days of binocular rivalry training. By the third day, the fluctuation of the LvsR values across the three sets of binocular rivalry tasks stabilized within the 10% criterion; thus the participant was deemed eligible to start the subsequent formal experiment. (b) For this participant, the changes of LvsR values for three blocks of binocular rivalry stabilized within 10% on the fourth day. The black dashed line indicates ideally balanced ocular dominance when the LvsR value equals 1. [file PCHJ-14-84-s001.docx]

**Supplemental Material**

Jue Wang, Xin He, Min Bao

**Supplemental Results**

**Distribution of phase durations**

We examined the distribution of phase durations for the stable percepts during rivalry, categorized by the testing stimuli. The distribution of phase durations has been found to approximate a two-parameter (*ρ*, *λ*) gamma distribution in the previous studies (Levelt, 1967; Lunghi et al., 2013). The formula is as follows:

$g\left( x \right)= \frac{\lambda^{\rho} x^{\rho-1}}{\Gamma(\rho)} e^{－\lambda x}$ (1)


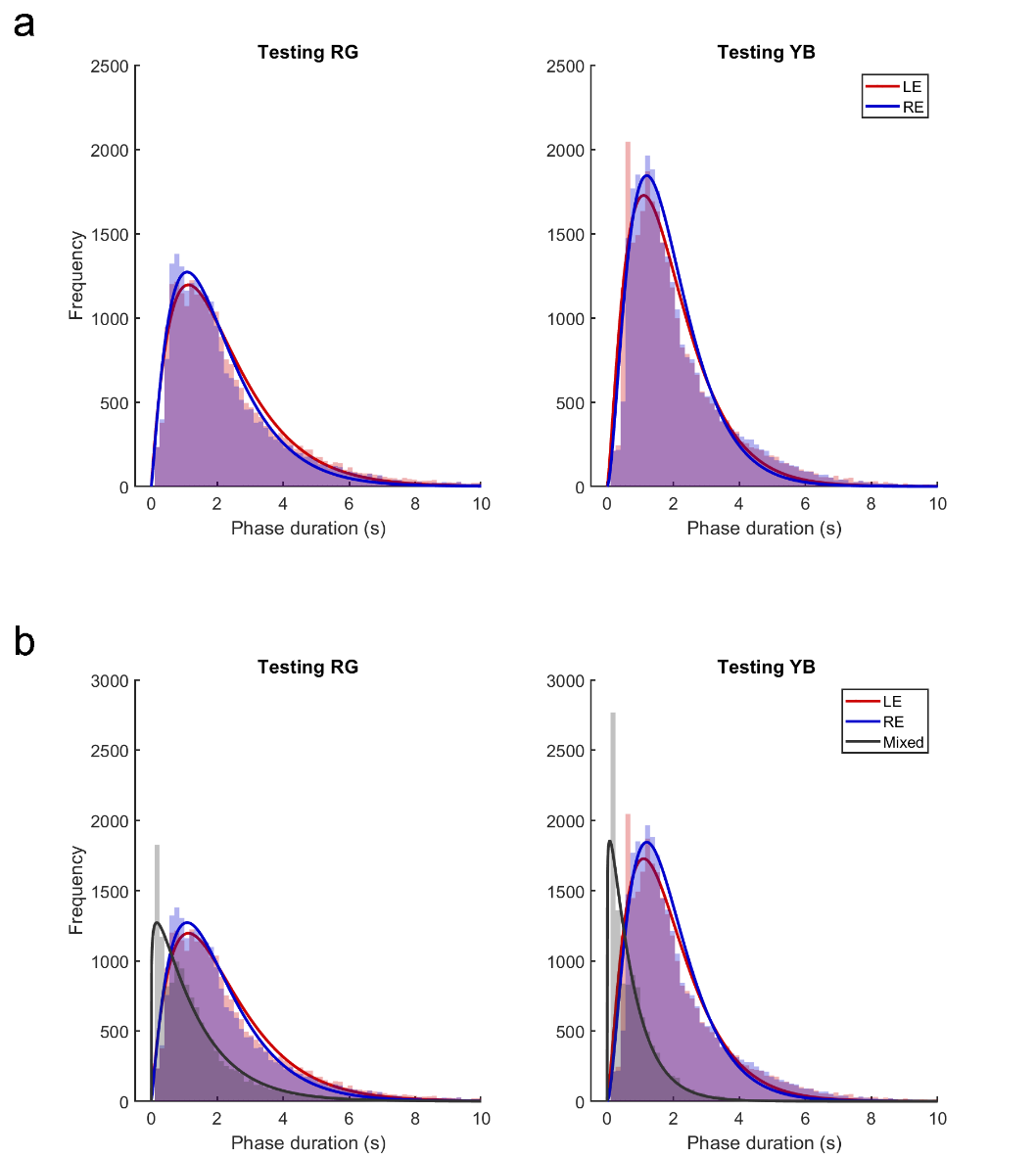
where *Γ* is the gamma function, *ρ* is the shape parameter, and *λ* is the scale parameter. The fits to the phase duration distributions for the left eye, right eye and mixed percepts are shown in Fig. S1. The quality of rivalry data for the two testing stimuli was fairly close to each other.

Fig. S1 Phase duration distributions are well fitted by a two-parameter gamma distribution of the form given in Equation (1), categorized by the testing stimuli. The left panel shows the phase duration distribution when using the R-G testing stimuli, and the right panel displays the phase duration distribution when using the Y-B testing stimuli. Red, blue and black curves show the fits to the phase duration distributions for the left eye, right eye and mixed percepts, respectively.

**Data from two representative participants in the binocular rivalry task training**

To assess ocular dominance for each participant, an eye ratio index named LvsR was calculated by the formula (*T_LE_* + *T_mix_*/2) / (*T_RE_* + *T_mix_*/2). In the formula, *T_LE_*, *T_RE_* and *T_mix_* represented the summed phase durations for perceiving the stimulus presented to the left eye, right eye, and mixed percepts, respectively. Following our previous work (Bai et al., 2017; Bao et al., 2018), we used this index to assess the stability of binocular rivalry performance in the practice phase and determine the dominant eye. Participants were allowed to advance to the formal experiment if the maximum LvsR value among the three tests did not exceed 110% of the minimum one
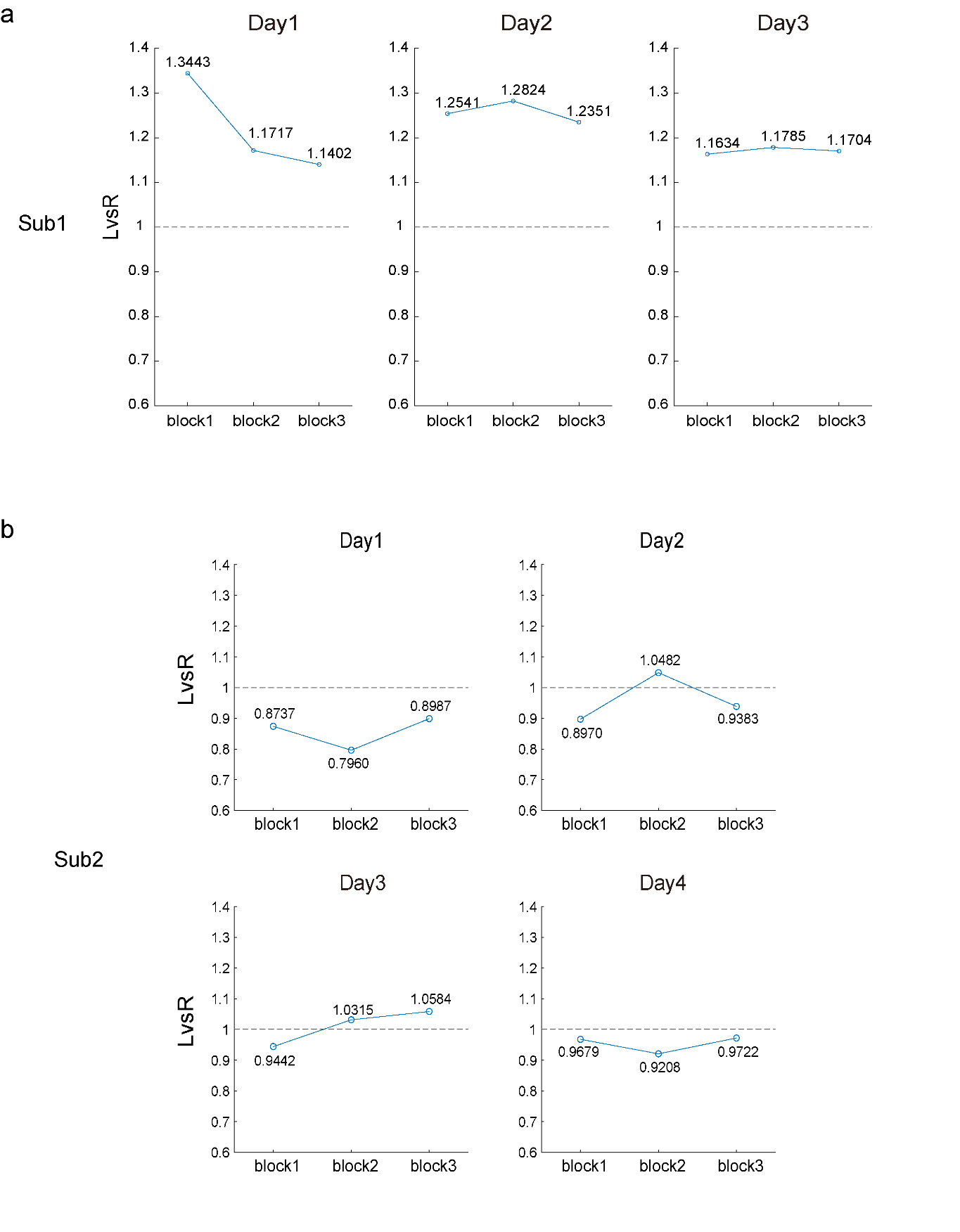
(which suggested the binocular rivalry performance had been relatively stable).

Fig.S2 Ocular dominance (LvsR) from two representative participants during the binocular rivalry task training. (a) Participant 1 underwent three days of binocular rivalry training. By the third day, the fluctuation of the LvsR values across the three sets of binocular rivalry tasks stabilized within the 10% criterion, thus the participant was deemed eligible to start the subsequent formal experiment. (b) For this participant, the changes of LvsR values for three blocks of binocular rivalry stabilized within 10% on the fourth day. The black dashed line indicates ideally balanced ocular dominance when the LvsR value equals 1.

References

Bai, J., Dong, X., He, S., & Bao, M. (2017). Monocular deprivation of Fourier phase information boosts the deprived eye's dominance during interocular competition but not interocular phase combination. *Neuroscience*, *352*, 122–130. <https://doi.org/10.1016/j.neuroscience.2017.03.053>

Bao, M., Dong, B., Liu, L., Engel, S. A., & Jiang, Y. (2018). The best of both worlds: Adaptation during natural tasks produces long-lasting plasticity in perceptual ocular dominance. *Psychological Science*, *29*(1), 14–33. <https://doi.org/10.1177/0956797617728126>

Levelt, W. J. (1967). Note on the distribution of dominance times in binocular rivalry. *British Journal of Psychology*, *58*(1), 143–145. <https://doi.org/10.1111/j.2044-8295.1967.tb01068.x>

Lunghi, C., Burr, D. C., & Morrone, M. C. (2013). Long-term effects of monocular deprivation revealed with binocular rivalry gratings modulated in luminance and in color. *Journal of Vision*, *13*(6), 1. <https://doi.org/10.1167/13.6.1>
